# Supplementary material for: Behaviour and welfare assessment of autochthonous slow-growing rabbits: The role of housing systems
Source: PLoS One. 2024 Jul 18;19(7):e0307456. doi: 10.1371/journal.pone.0307456 (PMC11257336; doi:10.1371/journal.pone.0307456)
Supplement: S1 File — (DOCX) [file pone.0307456.s001.docx]

**Supplementary material**

**Correlation matrix between rabbit behaviors**

**Figure S1.** Heatmap representing correlations between behaviors of rabbits. The Heatmap is symmetric. Each cell of the heatmap represents pairwise Spearman correlation between the two parameters indicated in the respective row and column. Correlations that reached significance after Bonferroni correction are indicated with the corresponding Spearman correlation coefficient (rho).

**Tonic Immobility**

**Table S1.** Effect of population (Po), housing system (Sy) and age (A) on tonic immobility test attempts. Mean and standard deviation of the seconds are indicated.

| **Tonic immobility test** | **Population (Po)** | | **Housing System (Sy)** | | | **Age (A)** | | | | **p-value** | | | | | |
| --- | --- | --- | --- | --- | --- | --- | --- | --- | --- | --- | --- | --- | --- | --- | --- |
|  | GC | GM | S | G | M | T1 | T2 | T3 | T4 | Po | Sy | A | Po x Sy | Po x A | Sy x A |
| first | 4.67 $\pm$ 15.7 | 10.96 $\pm$ 27.08 | 12.85 $\pm$ 29.25 | 8.07 $\pm$ 22.49 | 2.53 $\pm$ 9.84 | 10.53 $\pm$ 31.93 | 6.20 $\pm$ 18.77 | 10.33 $\pm$ 22.72 | 4.21 $\pm$ 10.75 | 0.12 | 0.11 | 0.62 | 0.22 | 0.65 | 0.57 |
| second | 10.30 $\pm$ 23.04 | 12.76 $\pm$ 36.09 | 13.43 $\pm$ 34.58 | 12.73 $\pm$ 33.63 | 8.42 $\pm$ 20.90 | 6.83 $\pm$ 32.86 | 9.83 $\pm$ 22.11 | 14.53 $\pm$ 36.25 | 14.93 $\pm$ 28.41 | 0.65 | 0.72 | 0.68 | 0.82 | 0.16 | 0.23 |
| third | 2.97 $\pm$ 10.41 | 2.92 $\pm$ 12.40 | 0.77 $\pm$ 3.56 | 5.15 $\pm$ 16.84 | 2.92 $\pm$ 9.50 | 6.80 $\pm$ 18.63 | 2.53 $\pm$ 6.98 | 0 | 2.46 $\pm$ 10.62 | 0.98 | 0.23 | 0.13 | 0.83 | 0.68 | 0.85 |
| average | 5.98$\pm$8.80 | 8.88$\pm$14 | 9.02$\pm$13.61 | 8.65$\pm$12.86 | 4.62$\pm$7.56 | 8.05$\pm$15.10 | 6.18$\pm$8.69 | 8.28$\pm$12.99 | 7.20$\pm$9.47 | 0.176 | 0.179 | 0.90 | 0.13 | 0.16 | 0.42 |

Figure S2. Average duration of tonic immobility in GC and GM rabbits, depicting the effect of population, housing system, and time. The box and whisker plots illustrate the interquartile range, and the black lines indicate the median. The error bars extend from the box to the highest and lowest values. The diamonds indicate the outlier’s data. N=120 (N = 10/population/housing system/time).

**HL and oxidative stress**

**Table S2.** Effect of population (Po), housing system (Sy) and age (A) (days) on heterophil/lymphocyte ratio (H/L) and oxidative stress parameters. Mean and standard deviation are indicated.

|  | **Population (Po)** | | **Housing System (Sy)** | | | **Age (A)** | | | **p-value** | | | | | |
| --- | --- | --- | --- | --- | --- | --- | --- | --- | --- | --- | --- | --- | --- | --- |
|  | GC | GM | S | G | M | 100 | 120 | 150 | Po | Sy | A | Po x Sy | Po x A | Sy x A |
| H/L | 0.98$\pm$ 0.67 | 1.21$\pm$0.70 | 1.03$\pm0.78$ | 0.90$\pm0.30$ | 0.79$\pm0.52$ | 0.90  $\pm0.30$ | 1.29  $\pm0.73$ | 0.99  $\pm076$ | 0.26 | 0.07 | 0.24 | 0.74 | 0.06 | 0.90 |
| umolHClO.ml | 268.64$\pm$54.41 | 271.76$\pm22.90$ | 255.1$\pm31.15$ | 281.8$\pm14.43$ | 279.5$\pm54.21$ | 281.80$\pm14.43$ | 275.15$\pm59.96$ | 259.45$\pm22.28$ | 0.79 | 0.10 | 0.30 | 0.53 | 0.96 | 0.29 |
| UCARR | 398.76$\pm$ 135.35 | 394.96$\pm$111.91 | 419.45$\pm118.41$ | 325.10$\pm124.53$ | 410.15$\pm119.04$ | 325.1$\pm124.53$ | 412.5$\pm121.93$ | 417.1$\pm115.58$ | 0.91 | 0.11 | 0.12 | 0.47 | 0.58 | 0.10 |
