# Supplementary material for: Behaviour and welfare assessment of autochthonous slow-growing rabbits: The role of housing systems
Source: PLoS One. 2024 Jul 18;19(7):e0307456. doi: 10.1371/journal.pone.0307456 (PMC11257336; doi:10.1371/journal.pone.0307456)
Supplement: Attachment — Submitted filename: Response to Reviewers.docx [file pone.0307456.s002.docx]

**Response to Editor:**

*Dear Dr. Portugaliza*

Thank you for considering our manuscript. We appreciate the opportunity to revise and improve our work to better meet PLOS ONE's publication criteria. We will carefully review the feedback provided and make the necessary revisions accordingly. We are committed to addressing the points raised during the review process to enhance the quality and relevance of our research. We look forward to resubmitting the revised manuscript and hope that it will meet your expectations.

Regarding the additional requirements:

1- We apologize for any oversight regarding PLOS ONE's style requirements, including file naming conventions. We have reviewed and adjusted our manuscript to ensure compliance with these guidelines. Our aim is to adhere to all necessary formatting standards to facilitate a smooth review process. We appreciate your guidance and will ensure that future submissions align closely with PLOS ONE's style requirements.
2- In this trial, our methodology involved raising animals until they reached the appropriate age for slaughter, without directly conducting the slaughter process. Consequently, we lack specific information regarding (1) methods of sacrifice, (2) anesthesia and/or analgesia techniques employed, and (3) efforts undertaken to alleviate any potential suffering experienced by the animals.
3- The role the funders took in the study is now clearly stated as you indicated.
4- Upon acceptance of the paper, we are fully committed to providing the data in a public repository such as Zenodo, as requested. We recognize the importance of transparency and scientific rigor in research, and we are dedicated to ensuring that our data is readily accessible to the scientific community for verification and further analysis. Thank you for emphasizing the significance of this practice, and we assure you that we will comply with this requirement without any hesitation.
5- We have amended our manuscript to ensure that the ethics statement is appropriately located within the Methods section as per the journal's requirements.
6- Thank you for bringing this matter to our attention. We sincerely apologize for the oversight regarding the reference to the figure in our manuscript. Upon review, we have indeed removed the figure in question and have made the necessary adjustments in the text accordingly.
7- Captions for the Supporting Information files have been appended at the end of the manuscript, ensuring clarity and proper referencing.

**Reviewer #1**

*GENERAL COMMENT*

*The present work was aimed at evaluating the behaviour and some physiological indicators of stress in two Italian local breeds of rabbits reared in conventional and alternative housing systems. The topic of the present work is interesting, but the manuscript needs a revision to clarify some key points.*

*In my opinion the work would have been more impactful with the presence of a commercial hybrids used as control. This comparative approach would allow for a better understanding of how different genotypes respond to challenging conditions and could contribute to discussions on the sustainability and resilience of various livestock farming systems. It may be beneficial for future research to consider such comparisons to enhance the overall impact and relevance of the findings.*

*The study could benefit from additional recordings to offer a more comprehensive evaluation of the impact of housing systems on animal welfare. Specifically, the absence of lesion scoring is a notable limitation, as it hinders a thorough assessment of potential physical impacts on the animals. Furthermore, including basic data on live weight, growth rate, and feed efficiency would enhance the overall understanding of the animals' physiological responses and performance under different housing conditions. Incorporating these additional measures would contribute valuable insights to the study, providing a more holistic perspective on the welfare and productivity implications of diverse housing systems.*

*One notable limitation of this paper pertains to certain gaps in the materials and methods section. Specifically, there is a need for clarification regarding key information, such as the number of replicates per group, the total number of observed rabbits, and the sex of the animals. These details are crucial for a comprehensive understanding of the experimental design and the robustness of the study’s findings. Addressing these omissions would enhance the transparency and reliability of the research, providing readers and fellow researchers with essential insights into the experimental setup and the transferability of the results.*

**Response:**

Thank you for your thoughtful review and valuable feedback on our manuscript. We appreciate your recognition of the importance of the topic and your suggestions for improving the clarity and impact of our work.

Your suggestion regarding the inclusion of a commercial hybrid breed as a control group is indeed valuable. We agree that such a comparative approach would offer valuable insights into how different genotypes respond to various housing systems, but our goal was to focus on giving specific information about these two rabbit populations, investigating if there are differences in behavior and adaptability or if these animals can be assimilated as a whole.

We also acknowledge your point regarding the absence of lesion scoring which might be a limitation.

We acknowledge the significance of incorporating data on live weight, growth rate, and feed efficiency to gain a deeper understanding of the physiological responses and performance of rabbits across different housing conditions. However, our study aimed to concentrate on observing the behavioral and welfare alterations exerted on these animals by the farming system. Undoubtedly, a future study, perhaps including a commercial control, focused on growth performance will be our priority. Furthermore, we value your feedback concerning gaps in the materials and methods section. We acknowledge the importance of providing clear and detailed information regarding experimental design and parameters. Consequently, we have revised the manuscript to address these omissions, ensuring transparency and reliability in our reporting.

Overall, your feedback will greatly contribute to improving the quality and impact of our research. We are committed to addressing your suggestions and enhancing the rigor and relevance of our work. Thank you once again for your valuable input.

SPECIFIC COMMENTS

**ABSTRACT**

- *L13: from this line the authors introduce the term “populations”. It should be clarified if these are two populations of the same breed or if they are two different breeds.*

**Response:**

The term "population" has been chosen to differentiate the animals because, despite these rabbits not being officially recognized as a breed, they exhibit unique characteristics and are widely spread and recognized in Italy, having a strong connection with the territory. To clarify the utilization of the term 'population' mentioned herein, we have added this sentence in the abstract: “These rabbits are not yet officially recognized as breeds, but they are commonly used in Italy for meat production and represent a distinctive phenotype and local heritage among farmers and consumers.”

- *L13: The authors could introduce the acronym for the two populations.*

**Response:**

We did not introduce acronyms in the abstract, following the guidelines of the journal, which suggest avoiding their use in this section.

- *L16: before the results it would be useful to report some other information regarding materials and methods, i.e., number of animals and replicates per group, duration of the trial, age of the animals.*

**Response:**

Thank you for your feedback. We appreciate your suggestion regarding the inclusion of additional information. We have incorporated details such as the number of animals and replicates per group, the duration of the trial, and the age of the animals before presenting the results. This will ensure clarity and completeness in our methodology description.

- *L25: did the rabbits reach the sexual maturity during the trial?*

**Response:**

Yes, the rabbits reached sexual maturity during the trial, and their age was specified in the text.

- *L26-28: is this the first study that demonstrate the correlation between agonistic behaviours and corticosterone levels?*

**Response:**

The correlation between corticosterone levels and agonistic behaviors has been well established since the study by Girolami et al. (1996). We have added this reference in the discussion section and in the references list.

**INTRODUCTION**

- *L41-43: this sentence does not seem particularly useful. It could be cancelled.*

**Response:**

We have removed this sentence as suggested.

- *L54-55: there is recent scientific literature regarding the characteristics of alternative rearing systems including information on cage/pen dimensions and environmental enrichment for group-housed rabbits. It could be useful to include a brief description and cite pertinent articles.*

**Response:**

Thank you for bringing attention to the recent scientific literature on alternative rearing systems for rabbits. It's indeed crucial to consider factors such as cage/pen dimensions and environmental enrichment when discussing the welfare of group-housed rabbits. We have included a brief overview of these findings in this section providing citations to support our points. This will help us better understand the optimal conditions for rabbit rearing and enhance the quality of our work. We have added the following sentences in the introduction, and we have cited the pertinent articles as suggested by the reviewer: “Alternative rearing systems for rabbits encompass a variety of approaches designed to improve animal welfare and optimize production efficiency. Central to these systems are considerations such as cage/pen dimensions and environmental enrichment, which play pivotal roles in promoting the physical and psychological well-being of group-housed rabbits [5]. Cage or pen dimensions in alternative systems often prioritize spaciousness to allow for increased mobility and social interaction among rabbits [6]. Additionally, environmental enrichment strategies such as providing tunnels, platforms, or chew toys offer opportunities for mental stimulation, physical exercise, and natural behaviors such as burrowing and exploring. These elements are essential for ensuring the overall health and happiness of group-housed rabbits, contributing to their quality of life and productivity in alternative rearing systems [7].”

- *L63-65: it is clear, but why did you choose two populations of the same breed? It should be motivated in the introduction because in terms of impact it would be more useful to compare two different local breeds or a local breed with a commercia genotype. This is what I would have expected reading this sentence.*

**Response:**

Thank you for your input. We selected these two populations because they are representative of the local territory, and our aim was to identify potential differences between them in relation to the type of farming system. While comparing different local breeds or a local breed with a commercial genotype could offer valuable insights, our focus was specifically on assessing variations within these specific populations under different breeding conditions. We will consider your suggestion for providing additional context in the introduction to clarify our rationale for this choice.

- *L67: it could be useful to introduce the name of the two populations and their acronyms here.*

**Response:**

Thanks for this suggestion. We have added the names and the acronyms for the two populations as suggested.

**MATERIALS AND METHODS**

- *L77-86: It is necessary to provide clear information regarding the number of replicates per group (cages/pens), and the sex of the animals.*

**Response:**

Thank you for this suggestion. To provide clarity on the methodology, we have incorporated all the requested information from the reviewer into Section 2.1 'Animals and Housing'.

- *L79-83: For the housing systems I would use acronyms that can be easily remembered and interpreted, e.g., Single, Group, Mixed.*

**Response:**

We fully agree with the reviewer that 'Single,' 'Group,' 'Mixed' are acronyms that can be easily remembered and interpreted. Therefore, from this point forward, we have replaced the acronyms 'S,' 'G,' 'C' with 'Single,' 'Group,' 'Mixed'.

- *L91: What exactly was the slaughter age?*

**Response:**

We have specified the exact slaughter age as suggested.

- *L91-93: Since the authors monitored the health (I think morbidity and mortality) I would expect a description of these results in the next section (Results).*

**Response:**

We conducted regular monitoring of various parameters throughout the trial to ensure the well-being of the animals involved, with the primary goal of promptly identifying and removing any potentially sick or distressed individuals to prevent unnecessary suffering. Notably, no sick rabbits were identified during the course of the trial.

A significant consideration in our experimental design was the decision to conclude welfare evaluations at 100 days of age. This decision was made proactively to mitigate the potential need to remove group animals due to aggressive behaviors, particularly as rabbits approached sexual maturity. While aggressive behaviors observed before the 100-day mark were not detrimental to the rabbits' welfare, we anticipated that such behaviors might escalate in severity post-sexual maturity. Consequently, continuation of the trial beyond this point could have necessitated the removal of group animals, which would have compromised the integrity of the study.

By focusing our investigation on the impact of reaching sexual maturity on rabbit behavior while ensuring their well-being and minimizing distress, we aimed to provide a comprehensive understanding of this aspect without subjecting the animals to undue harm or disruption. We believe that this rationale is now more clearly articulated and integrated into the conclusions of our study.

- *L96-97: Please specify the exact number of rabbits observed per group.*

**Response:**

We have specified the exact number of rabbits observed per group as suggested.

- *L98-100: This is a weakness of the work since it is widely known that rabbits are active during night. The authors should consider this aspect when discussing the results.*

**Response:**

Thank you for your comments, which have enabled us to clarify the circadian rhythm of the observed rabbits. To address this point, we have included the following sentences in the discussion section:

“Our study may have limitations considering that observations of animal behaviors were conducted during daylight hours. European wild rabbits are primarily active at night, particularly at dusk and dawn, and they typically reside and rest during the daytime in their warrens (Villafuerte et al., 1993). However, it has been demonstrated that external factors such as noise or scheduled feeding during the light period can shift their activity patterns, making them predominantly diurnal (Diez et al., 2013). Thus, our observations remain reliable regarding the behavior of these animals, and our results can be generalized for rabbits held in captive conditions”.

- *L175-184: Did you perform a check of data distribution? Which distribution was set for the model?*

**Response:**

Yes, we performed a check of data distribution. Normality of data distribution was assessed using the Shapiro-Wilk test. For the Generalized Linear Model (GLM) used to explore the relationships between the rabbits' behaviors and CORT levels, we assumed a gamma distribution with a log link function. This distribution was chosen based on its suitability for modeling non-normally distributed continuous data. The log link function was selected to ensure that the model predictions remained on the positive scale, consistent with the nature of CORT data. We have added this information in section 2.6 “Statistical analysis”.

**RESULTS**

- *L195: The acronyms should appear in the abstract and then the first time you define the population in the main text (L67).*

**Response:**

We have managed the acronyms for the populations as suggested. However, we did not introduce acronyms in the abstract, following the guidelines of the journal, which suggest avoiding their use in this section.

- *L199-234: Reporting p-values and the term “significant” in redundant. It would be more useful to report some relative numerical variations among groups.*

*L236-260: For a more detailed and informative presentation of the results, providing relative numerical variations among groups when significant would enhance the clarity and precision of the findings. This approach can help readers better understand the magnitude and direction of the observed effects within and across different groups, contributing to a more comprehensive interpretation of the study outcomes.*

**Response:**

Accordingly, we have removed the term 'significant' in this section. We agree that adding numerical variations of frequencies for certain behaviors could enhance the understanding of the observed effects. We have included this information for specific behaviors in the comparison between the two populations (GM and GC). However, for the comparison involving three groups such as the housing systems, we believe that additional numerical details might confuse the reader. Since this information is already clearly presented in Table 3, we opted against including it in the text to avoid redundancy and maintain the clarity and readability of the manuscript.

**DISCUSSION**

*The structure of the discussion should be changed. It would be better to discuss the results according to the main effects analyzed (population, housing systems and age).*

*Probably, the most interesting interaction is that between housing system and age due to the presence of the Mixed system. The discussion regarding this interaction should be expanded.*

*Three other key aspects should be included and considered in the discussion: 1) the sex of the animals; 2) the number of animals observed (if I understood correctly, it was not that high); 3) the period of observation (limited to direct observations during daylight).*

**Response:**

Thank you for these suggestions. We have revised the structure of the discussion to focus on the main effects analyzed, namely population, housing systems, and age. Additionally, we have expanded the discussion regarding the interaction between housing system and age. Furthermore, we have incorporated three key aspects into the discussion: 1) the sex of the animals, 2) the number of animals observed, and 3) the period of observation. Thank you for your valuable suggestions, and we believe these revisions have strengthened the discussion section.

- *L266-269: there are more pertinent papers that report the effect of the housing system on rabbits’ behaviour and welfare.*

**Response:**

Thank you we have provided a more pertinent reference.

- *L300-303: these results are not reported in the previous section. Please implement the section Results before their discussion. Furthermore, there is no information regarding morbidity and mortality that are crucial for an evaluation of the animal welfare state.*

**Response:**

Thank you for your observations. We have incorporated the missing results into the Results section 3.2, 'Behavioral Observations'.

- *L305-306: This is not a clear discussion of the results. Please, try to provide a more detailed hypothesis.*

**Response:**

Thank you for your feedback. We acknowledge the need for a clearer discussion of the results in this section. Regarding comfort activities, our observation of a significant population effect, with GC rabbits showing a higher tendency for self-scratching and self-grooming compared to GM rabbits, suggests the possible presence of genetic differences between the two populations influencing their behavioral patterns. We hypothesize that these differences may stem from selective breeding practices or environmental factors specific to each population. Further investigation into the genetic and environmental factors contributing to these behavioral differences could provide valuable insights into the underlying mechanisms shaping rabbit behavior. We have added these sentences in the discussion section.

- *L340-348: Perhaps it should be better referring to a correlation analysis rather than to a GLM that is the procedure used for testing the correlation.*

**Response:**

Thank you for your suggestion. While we appreciate the consideration of correlation analysis, we believe that employing a Generalized Linear Model (GLM) was more appropriate for our analysis as it allows for the examination of multiple variables simultaneously and provides more comprehensive insights into the relationships between behaviors and physiological stress responses. GLM enables us to assess the significance of these associations while controlling for potential confounding factors, which is crucial in our study context.

**CONCLUSIONS**

*L362-368: this is a summary of the results.*

*L368-371: honestly this conclusion seems very generic and not fully supported by the results since each system showed strengths and weaknesses.*

**Response:**

Thank you for your comments. We have completely and carefully revised the conclusion in response to your feedback, aiming to address the strengths and weaknesses observed in each housing system while providing a more nuanced interpretation of the results. We appreciate your guidance and believe that the revised conclusion better reflects the complexities of the findings and their implications.

**Reviewer #2**

**Broad comments**:

*The authors are based in a country where rabbit production is very important. Thus, this subject is relevant for the field and address a welfare issue, which are housing systems, which greatly affect rabbits’ welfare. Behavioral indicators were well evaluated and described, including the studied repertoire and their approach. However, the corticosterone evaluation seems not to be adequate for the specie. There is little information on literature regard their corticosterone circadian rhythm to state that the studied animals were more stressed based in this single measure. Moreover, just a single-time measure was performed, which does not give us an overview of the circadian rhythm. I am not convinced that the hair corticosterone is informing something. Based in other reviewers’ comments, may it would a good idea to remove your corticosterone data from your manuscript and describe and explore more your behavioral data, despite the fact that you compare single housing with group housing for a social animal. In a “perfect” world, you must have a “perfect” environment, and explore and measure how an environment with more space, ground contact, and complex social network may affect the individuals. Moreover, there are important information missing. Please, find my specific comments bellow.*

**Response:**

We appreciate the reviewer's thoughtful evaluation of our manuscript. While we understand the concerns regarding the adequacy of corticosterone evaluation for the species and the limitations of a single-time measure, we believe that incorporating corticosterone data alongside behavioral indicators provides a more comprehensive understanding of the animals' stress responses. It's important to note that we conducted repeated measurements over time (T1, T2, T3, T4) rather than a single measure, which allows for a more detailed assessment of stress dynamics. Although there may be limited literature on the corticosterone circadian rhythm in this species, our aim was to explore potential associations between physiological stress responses and behavioral patterns. We agree that exploring and measuring the effects of different environmental conditions, such as single housing versus group housing, is crucial for understanding the well-being of social animals. However, we believe that including corticosterone data allows for a more complete examination of stress responses in conjunction with behavioral observations. Moreover, hair corticosterone offers insights into long-term stress exposure, complementing the information obtained from saliva samples.

Specific comments:

- *Line 59-60: Please, insert reference regarding aggressive behavior*.

**Response:**

Thank you for your suggestion, we have added the following reference:

Rödel, H. G., & von Holst, D. (2009). Features of the early juvenile development predict competitive performance in male European rabbits. Physiology & behavior, 97(3-4), 495-502.

- *Line 70: The ethogram is just a tool.*

**Response:**

We appreciate the reviewer's feedback, and we have accordingly revised the sentence**.**

- *Line 79-86: Please, standardized the measure units (mm, m2, animals per square meter or <40kg per m², etc). In order to make more comparable, I suggest standardize for kg/m²).*

**Response:**

Thank you, we simplified using only m^2^ to indicate the dimensions of the pen and kg/m² for animal density as commonly used by other authors.

- *Line 89: “+15/+28 °C” Do you mean 15-25°C?*

**Response:**

We intended to specify a range between 15°C and 28°C.

- *Line 91: ad libitum*

**Response:**

Thank you, we have corrected *ad libitum.*

- *Line 97: used instead of employed.*

**Response:**

We have modified the word as per the reviewer's suggestion.

- *Line 98: How did you define these ages for behavioral measures? You must explain the reasons to choose these specific ages. Same comments for the time of the day (9-11 a.m.; 2-4 p.m.).*

**Response:**

Thank you for the suggestions. We have addressed these points in the 'Behavioral Analysis' section 2.2.

- *Line 99: Are rabbits inactive during nighttime?*

**Response:**

Thank you for your question, which has enabled us to clarify the circadian rhythm of the observed rabbits, as also requested by reviewer 1. To address this point, we have included the following sentences in the discussion section:

“Our study may have limitations considering that observations of animal behaviors were conducted during daylight hours. European wild rabbits are primarily active at night, particularly at dusk and dawn, and they typically reside and rest during the daytime in their warrens (Villafuerte et al., 1993). However, it has been demonstrated that external factors such as noise or scheduled feeding during the light period can shift their activity patterns, making them predominantly diurnal (Diez et al., 2013). Thus, our observations remain reliable regarding the behavior of these animals, and our results can be generalized for rabbits held in captive conditions.”

- *Line 110: The authors’ name or reference number is wrong. Number 3 reference is Munari et al., and Mugnai et al is reference number 6.*

**Response:**

Thank you so much for your observation. We have corrected the reference number accordingly.

- *Line 112: That means that you measure only the frequency of the behaviors and not the duration of it. Am I right? If so, please make it clear in the manuscript.*

**Response:**

We have clarified this point by adding the following sentence: “The behaviours were observed and registered as a frequency measure not considering duration.”

- *Line 114: This is the first time in the manuscript that is presented this test. There is none description or mention about how this test could help the authors regarding their research question. What time in the day this test was carried out? Prior or previous behavioral evaluation? Was the operator who performed the test the same as the one who conducted the behavioral evaluation? This is an important information, since the presence of the person who did the test may interfere on rabbits’ behavior.*

**Response:**

Thank you for your feedback. We fixed the citation. We also acknowledge the importance of providing comprehensive information regarding the rationale and significance of the tests conducted. We have included a detailed explanation of this test and how the resulting data contribute to addressing our research question. We agree that this clarification will enhance the transparency and comprehensiveness of our study, enabling readers to better understand the experimental design and its relevance to our research objectives. The tonic immobility test is now introduced in the introduction section and has been better described in the materials and methods section.

- *Line 119: Are you sure this is the correct reference? The cited manuscript did an open-field test and did not mention any immobility test. Moreover, I did not find any information regarding this test in the manuscript. The authors should include why you performed this test and how these data could contribute to your research question.*

**Response:**

Thank you for bringing this to our attention. We have rectified the reference error, and it is now accurately cited.

- *Line 141: what time in the day did you collect saliva? It was early morning, late morning, early afternoon. This information is crucial to understand the circadian rhythm of corticosterone.*

**Response:**

The time of day at which we collected the saliva samples has been specified as requested in section 2.5.

Previous research focusing on the diurnal rhythm of salivary corticosterone concentration in rabbits suggests that their stress hormone levels fluctuate throughout the day, with a significant increase between 12:00 and 15:00 (Kadlekova et al. 2023) this information has been integrated in the discussion.

- *Line 149-166: I am concern about your measurement in hair cortisol. The protocol seems adequate; however, the matrix is not the most indicate. We have scientific evidence in other species that the hair concentration can differ depending on the color of the hair in dogs (Benett and Hayssen, 2010); age and color (Binz et al., 2018); body regions and color in cows (Burnett et al., 2014); body regions, age, hair color, and season in cows and in pigs (Heimbürge et al., 2020), among others. Moreover, your reference number 17 and 18 in the text are different from your reference list. You cited Meyer et al as 17 but in the reference list is number 18. Please, take a thorough look in all your references links.*

**Response:**

We understand your concern regarding the measurement of hair cortisol. While we acknowledge the potential influence of various factors such as hair color, age, body regions, and seasonal variations, it's worth noting that the coat of these rabbits is uniform and not like dogs or cows (Benett and Hayssen, 2010; Burnett et al., 2014). Despite these considerations, we still believe that hair cortisol assessment provides valuable insights into chronic stress levels. Its evaluation is important for providing indications of chronic stress.

- *Line 232: Could you mention here examples of “potential negative effects associated with sexual maturity”*

**Response:**

We have incorporated examples into the text as requested.

- *Line 267-268: I did not understand this statement here since you did not provided an environmental enrichment for the rabbits.*

**Response:**

We understand your observation. "Group housing" can indeed be considered as a form of "environmental enrichment" for rabbits compared to single cages. The concept of "group housing" offers a range of benefits beyond those of single cages, including social interaction, physical exercise, and mental stimulation. Furthermore, considering the "mixed group" dynamics, which reflects a more realistic social setting, adds another level of complexity that can positively contribute to the rabbits' welfare. Therefore, our intention was to compare traditional systems like single cages with more advanced approaches like "group housing," while also considering the physiological needs of rabbits, including the formation of mixed groups. I hope this clarifies my intent.

- *Line 288: “…because rabbits are a social species”*

**Response:**

Thank you for the suggestion. We have incorporated it into the text.

- *Line 357: here is the major example of how your stress data are questionable. How a social animal, selected from generations and generations to be social, could be more stressful when group housed?*

**Response:**

It's understandable to question the notion that social animals could experience increased stress when housed in groups, given their evolutionary predisposition towards social interactions. However, it's important to note that these animals are often raised and live in artificial rather than natural conditions. These rabbits are far from intensive hybrids genetically selected for intensive farming. In a controlled environment, social interaction can be influenced by a range of variables that may not necessarily reflect the social dynamics occurring in the wild. For instance, competition for resources such as food and living space can be exacerbated by the artificial scarcity of available resources in breeding environments.

Furthermore, the lack of opportunities for animals to engage in their natural behaviors can add further layers of stress. The need to adapt to a human-built environment rather than a natural one can pose additional challenges for social animals, influencing their psychological and behavioral well-being.

Therefore, while social interaction is undoubtedly crucial for the welfare of social animals, it's important to also consider the artificial context in which they live and are raised when assessing their stress levels in a group setting.
