# Supplementary material for: Behaviour and welfare assessment of autochthonous slow-growing rabbits: The role of housing systems
Source: PLoS One. 2024 Jul 18;19(7):e0307456. doi: 10.1371/journal.pone.0307456 (PMC11257336; doi:10.1371/journal.pone.0307456)
Supplement: Attachment — Submitted filename: response to reviewer.docx [file pone.0307456.s003.docx]

**Reviewer #2: Broad comments:**

Thank you very much for your positive feedback and for acknowledging our efforts in addressing the comments. We appreciate your thorough review and are grateful for the opportunity to improve our work.

We would like to address your question by explaining our choice of measuring corticosterone in both saliva and hair. We understand your concern about the importance of circadian rhythms, which is why we decided to focus on both saliva and hair corticosterone. Our approach involves collecting and studying saliva corticosterone to provide measurements of acute stress. Since we always collected samples at the same time in the morning, this measurement reflects the stress from the previous night when the rabbits are more active.

We acknowledge that our sampling may not perfectly reflect circadian variations. However, we believe that this method could provide valuable indications of acute stress, and, by studying hair corticosterone, we can also investigate chronic stress. Repeated measurements over time (T1, T2, T3, and T4) allow us to evaluate the progression of stress levels in these animals. Consequently, in line with your comments, we have included the limitations regarding circadian rhythms in the discussion section of our manuscript to clarify our methodological choices and discuss the relevant constraints.

“**One limitation of our study is that our sampling method does not fully account for the circadian variations in corticosterone levels. Corticosterone levels naturally fluctuate throughout the day, and our fixed sampling time may not capture these dynamic changes accurately. As a result, while our morning saliva samples reflect the stress levels from the preceding night, they may not provide a comprehensive view of the fluctuations in corticosterone that occur over a 24-hour period.”**

Regarding the concern about uniform hair coat color, we agree that while it is beneficial, it does not ensure a constant and standardized deposition of corticosterone in the hair. We have included these limitations in the discussion section of our manuscript.

“**We acknowledge that a limitation of using corticosterone in hair as a biomarker for chronic stress is the individual differences in hair coloration. Even within a population of rabbits with uniform hair color, there may be individual variations that require further investigation.**”

Regarding environmental enrichment for social animals in group housing, it is crucial to address the need for appropriate environmental stimuli to meet behavioral needs, particularly when animals are housed together. In Europe, there is a growing movement towards abolishing single cages in favor of group housing for social animals. This shift reflects increasing recognition that group housing constitutes a substantial form of environmental enrichment, as recognized by both national and European regulations. For example, the European Union Directive 2008/120/EC on the protection of rabbits specifically mandates that rabbits must be housed in compatible groups unless specific reasons justify individual housing. This regulation acknowledges that group housing allows rabbits to engage in natural social behaviors, promoting better welfare compared to solitary or crated housing. This approach is in line with evolving regulatory frameworks and societal expectations aimed at advancing animal welfare standards across Europe.

Finally, regarding the last suggestion, the welfare challenges observed in rabbits raised in group housing settings primarily stem not from genetic factors but from the type of farming system itself, which may not be well-suited for rabbits, especially robust breeds like the Grey rabbits of Piedmont. This indigenous Italian breed has historically been raised in both cage-based and more extensive farming systems, known for their resilience and adaptation to local conditions rather than intensive cage farming. Our research focuses on addressing increasing consumer demand and European Union regulations aimed at phasing out cage systems in animal farming. These rabbits naturally exhibit behaviors such as dominance and aggression among males, complicating their transition to group housing arrangements. To mitigate these challenges, we propose a two-phase farming approach detailed in our paper. This approach considers their natural social dynamics and temperament, aiming to ensure their well-being while meeting market demands. Furthermore, due to their medium to slow growth rate compared to intensive hybrid breeds, the Grey rabbits of Piedmont require longer farming periods. This emphasizes the importance of adopting sustainable and welfare-oriented farming practices that cater specifically to their unique needs and behaviors.

**Specific comments:**

Line 17-18: “… 294 weaned males with 35 days old were divided…”

**Corrected as suggested**

Line 64: “… and psychological welfare of group-housed rabbits.”

**Corrected as suggested**

Line 101-110: Please, standardize you units. Density for Traditional single cage is expressed as animals per square meter and Group farming is expressed as kg per square meter. Mixed pilot systems have both units, kg/m² and animals/m². I already asked this during my first review.

**Thank you for your feedback. We have made the necessary corrections, and now all density values are uniformly expressed in kg/m² as requested.**
